# Serum microRNAs as Tool to Predict Early Response to Benralizumab in Severe Eosinophilic Asthma

**DOI:** 10.3390/jpm11020076

**Published:** 2021-01-28

**Authors:** José A. Cañas, Marcela Valverde-Monge, José M. Rodrigo-Muñoz, Beatriz Sastre, Marta Gil-Martínez, Raquel García-Latorre, Manuel J. Rial, Aida Gómez-Cardeñosa, Mar Fernández-Nieto, Erwin J. Pinillos-Robles, María J. Rodríguez-Nieto, Nicolás González-Mangado, Joaquín Sastre, Victoria del Pozo

**Affiliations:** 1 Department of Immunology, IIS-Fundación Jiménez Díaz, 28040 Madrid, Spain; jose.canas@fjd.es (J.A.C.); jose.rodrigom@quironsalud.es (J.M.R.-M.); bssastre@fjd.es (B.S.); marta.gilm@quironsalud.es (M.G.-M.); raquel.garcial@quironsalud.es (R.G.-L.); 2 CIBER de Enfermedades Respiratorias (CIBERES), Instituto de Salud Carlos III, 28029 Madrid, Spain; JSastre@fjd.es; 3 Allergy Unit, Hospital Universitario Fundación Jiménez Díaz, 28040 Madrid, Spain; marcela.valverde@quironsalud.es (M.V.-M.); manuterial@gmail.com (M.J.R.); aida.gomez@fjd.es (A.G.-C.); MMFernandez@fjd.es (M.F.-N.); 4 Pulmonology Unit, Hospital Universitario Fundación Jiménez Díaz, 28040 Madrid, Spain; erwin.pinillos@quironsalud.es (E.J.P.-R.); MJRodriguezN@fjd.es (M.J.R.-N.); NGonzalez@fjd.es (N.G.-M.)

**Keywords:** benralizumab, biologic treatment, microRNA, severe eosinophilic asthma

## Abstract

Severe eosinophilic asthma poses a serious health and economic problem, so new therapy approaches have been developed to control it, including biological drugs such as benralizumab, which is a monoclonal antibody that binds to IL-5 receptor alpha subunit and depletes peripheral blood eosinophils rapidly. Biomarkers that predict the response to this drug are needed so that microRNAs (miRNAs) can be useful tools. This study was performed with fifteen severe eosinophilic asthmatic patients treated with benralizumab, and serum miRNAs were evaluated before and after treatment by semi-quantitative PCR (qPCR). Patients showed a clinical improvement after benralizumab administration. Additionally, deregulation of miR-1246, miR-5100 and miR-338-3p was observed in severe asthmatic patients after eight weeks of therapy, and a correlation was found between miR-1246 and eosinophil counts, including a number of exacerbations per year in these severe asthmatics. In silico pathway analysis revealed that these three miRNAs are regulators of the MAPK signaling pathway, regulating target genes implicated in asthma such as *NFKB2*, *NFATC3*, *DUSP1*, *DUSP2*, *DUSP5* and *DUSP16*. In this study, we observed an altered expression of miR-1246, miR-5100 and miR-338-3p after eight weeks of benralizumab administration, which could be used as early response markers.

## 1. Introduction

Asthma is a chronic inflammatory disease of the airways that affects more than 300 million people worldwide [1]. This pathology causes shortness of breath, chest tightness, wheezing and cough, and presents a strong inflammatory component related to T2 immune response [2]. This disease exhibits an elevated heterogeneity and variability, which means that it is an ineffective asthma control in many cases. Moreover, its great economic cost on the health system makes it a big problem for government institutions.

Severe asthma comprises a small group of asthmatic individuals, between 5–10% of people with asthma who have a higher risk of severe exacerbation, clinical worsening and poor control. Among these patients, an estimated 40–60% have eosinophilic airway inflammation, which is associated with severe asthma, despite high-inhaled corticosteroids (ICS) and long-acting β2-agonists (LABAs) therapies [3]. According to the European Respiratory Society (ERS) and American Thoracic Society (ATS), severe asthma requires high doses of ICS, plus a second treatment and/or systemic corticosteroids to aver it [4]. This fact makes traditional treatments ineffective as they have to choose other alternative therapies, such as biological treatments.

Biological drugs are humanized monoclonal antibodies that block cytokines or receptors of cytokines, improving symptoms in asthmatic patients [5]. Specifically, in severe eosinophilic asthma, these drugs decrease the eosinophil percentage from peripheral blood and tissues, with the most used being: two anti-IL-5 drugs (reslizumab and mepolizumab) and one against the IL-5α receptor (benralizumab), depending on the clinical characteristics of patients. Benralizumab is a humanized afucosylated monoclonal antibody (IgG1κ) that binds with high affinity to the IL-5α receptor on the surface of human eosinophils and basophils [6]. This drug induces rapid and complete depletion of blood eosinophils through antibody-dependent cell-mediated cytotoxicity (ADCC), significantly reducing the number of exacerbations and improving lung functionality of patients with severe asthma [7]. These results were observed in three different clinical trials that used this pharmacological approach, namely SIROCCO, CALIMA and ZONDA. These studies showed that the severe eosinophilic asthmatics can benefit by the action of this treatment, as seen in the reduction of their exacerbation rate, and the improvement observed in asthma control and quality of life, while also reducing the dose of the daily oral corticosteroids needed [8,9,10]. This new biological drug has also been investigated as a treatment for chronic obstructive pulmonary disease (COPD) patients with more than 3% of sputum eosinophils with at least one exacerbation, being the results promising for lung function improvement, but not having any effect in the exacerbation rate, only proving efficacy in the patients with eosinophilic inflammation [11]. Furthermore, two additional investigations with this treatment in COPD were depicted in TERRANOVA and GALATHEA studies [12]. Both clinical trials included COPD subjects with or without a high eosinophil count (≥220 per mm^3^) with frequent exacerbations, and used different dosages of benralizumab. From the TERRANOVA study, no effects were observed by this treatment for any of the traits, while GALATHEA showed similar patterns of no effect by this drug, with only a trend for improvement of exacerbations with the 100 mg/dose. Despite several studies demonstrating the efficacy and security of benralizumab [8,9], it is necessary to search for biomarkers that determine the early response of patients to this therapy, leading to the personalized choice.

Till now, some works have addressed this topic [13,14], but more studies must be performed. In this sense, microRNAs (miRNAs) can help to predict the response to benralizumab. These small non-coding RNAs have been used as biomarkers in multiple diseases, including asthma [15]. MiRNA deregulation in pathologic status not only affects several biologic processes, but they can also be used to characterize and diagnose patients [16], implying an approach towards personalized medicine. Combining all these premises, miRNAs could be useful tools to detect the early response of asthmatic individuals to benralizumab and offers the best therapy for the patient.

In this context, the main aim of this study is to search some miRNAs that could serve as biomarkers to detect an early response to benralizumab in severe eosinophilic patients. Additionally, these miRNAs could correlate with some clinical parameters, which would allow for the observation of patient improvement.

## 2. Materials and Methods

### 2.1. Patient Selection

Subjects diagnosed with asthma were recruited from Allergy and Pneumology units of Fundación Jiménez Díaz Hospital in Madrid, selecting 15 patients with severe eosinophilic asthma and 15 mild-moderate asthmatic subjects. All patients belong to the study of the Mechanisms involved in the Evolution and Genesis of Asthma (MEGA) project, which is a cohort of asthma patients with varying grades of severity [17]. Asthma severity has been diagnosed according to the classification of the Global Initiative for Asthma (GINA) [18]. Fifteen healthy individuals were recruited from the Allergy Department of Fundación Jiménez Díaz.

The inclusion criteria were the following: (i) acceptance to participate; (ii) asthma diagnosis following GINA 2019 criteria [18]; (iii) age between 18–75 years old; (iv) periodic medical examinations; (v) intravenous administration of benralizumab (30 mg) for severe uncontrolled eosinophilic asthmatic patients (blood eosinophil count ≥300 cells/μL) every four weeks for the first three doses, and then every 8 weeks thereafter; and (vi) patients with mild to moderate persistent asthma treated with a combination of ICS/LABAs. For healthy individuals, the only requirement was not having any episodes of asthma.

All subjects gave and signed their informed consent for inclusion before they participated in the study. This study was conducted in accordance with the Declaration of Helsinki, and the protocol was approved by the Ethics Committee.

### 2.2. Sample Collection

Serum was obtained by blood clotting in anti-coagulant free tubes and centrifugation at 3000 rpm for 10 min at 4 °C, and stored at −80 °C until used. Peripheral blood from severe patients was recovered at two-time points: before benralizumab injection and 8 weeks after the first doses of the biological drug.

### 2.3. MiRNA Isolation

MiRNAs were obtained from 200 μL of serum using miRNeasy serum/plasma advanced kit (Qiagen, Hilden, Germany), as the manufacturer described. Three synthetic miRNAs spike-ins (SP2, SP4 and SP5) were added to evaluate the optimal RNA extraction (miRCURY LNA RNA Spike-in kit, Qiagen).

### 2.4. MiRNA Reverse Transcription PCR (RT-PCR) and Semi-Quantitative Real Time PCR (qPCR)

Serum miARNs were obtained by a first step of cDNA retrotranscription using the miRCURY LNA RT Kit (Qiagen), following manufacturer’s protocol. Briefly, 4 μL of total RNA were mixed with a reverse transcription enzyme, and with other synthetic miRNAs: Spike-in (UniSp6) and cel-miR-39-3p, which were used for control of the correct retrotranscription to cDNA. The total volume was 10 μL. The reaction was performed in a Veriti 96 well Thermal Cycler (Applied Biosystems, Warrington, UK), for 60 min at 42 °C, then 5 min at 95 °C, and indefinitely at 4 °C. cDNA was stored at −20 °C until used.

Then, miRNA expression was evaluated by qPCR using miRCURY LNA SYBR Green PCR Kit (Qiagen) according to the manufacturer’s instructions. In summary, cDNA from serum miRNAs obtained in the previous phase was diluted 1:30 in RNase free water (Qiagen). Then, 3 μL of diluted cDNA were mixed with 2X miRCURY SYBR Green Master Mix and with 1 μL the suitable probes in a final volume of 10 μL. The probes used were: hsa-miR-1246, hsa-miR-1290, hsa-miR-451a, hsa-miR-144-3p, hsa-miR-144-5p, hsa-miR-5100, hsa-miR-4521, hsa-miR-320a, hsa-miR-320b, hsa-miR-185-5p, hsa-miR-21-5p, hsa-miR-146b-5p, hsa-miR-486-5p, hsa-miR-629-5p and hsa-miR-338-3p. These miRNAs were selected based on previous work [16]. Additionally, hsa-miR-191-5p, hsa-let-7a and cel-miR-39-3p were selected as endogenous controls (Qiagen). Additionally, hsa-miR-23a-3p and hsa-miR-451a were used as hemolysis controls. All samples were run in triplicate, and the reaction was performed in a Light Cycler^®^ 96 thermocycler (Roche, Basilea, Switzerland). The incubation program was carried out for 45 cycles of 95 °C during 10 s and 60 °C for 1 min. DNA melting was performed by heating at 95 °C for 5 s, then 65 °C for 1 min, and finally at 97 °C for 1 s. Samples were cooled for 10 s at 40 °C. Cycle threshold (Ct) values were analyzed with LightCycler^®^ 96 SW 1.1 (Roche) software. MiRNA expression was calculated using the 2^−ΔCt^ method [19], where: ΔCt = Ct_miRNA_ − X(Ct_hsa-miR-191-5p_ + Ct_has-let-7a_ + Ct_cel-miR-39-3p_).

### 2.5. In Silico Pathway Analysis

Pathway analysis of deregulated miRNAs were performed using the DIANA-miRPath v3.0 bioinformatic tool [20].

### 2.6. Statistical Analysis

The results are expressed as mean ± standard deviation (SD), or median and interquartile range (IQR). Normality was analyzed using the Shapiro-Wilk test. For continuous variables, parametric data comparison between non-paired groups was performed using an unpaired *t* test (compared groups have equal SD) and a *t* test with Welch’s correction (assumption of population may have different SD), and finally non-parametric and non-paired groups were compared using the Mann-Whitney test. Comparison between paired groups was done with a paired *t* test for parametric data and with a Wilcoxon matched paired test for non-parametric data. Additionally, Spearman’s (for non-parametric data) or Pearson’s (for parametric data) correlation were applied for comparison between miRNA expression levels (ΔCt) and some clinical parameters.

## 3. Results

### 3.1. Benralizumab Improves Asthma Symptoms

To develop this study, fifteen severe eosinophilic patients treated with benralizumab were recruited. Additionally, fifteen mild to moderate asthmatic individuals and fifteen healthy subjects were also included in the study. We observed that patients who received benralizumab were significantly older than mild–moderate asthmatics, although they did not present statistical differences in relation to gender (Table 1). It is worth noting that after eight weeks of treatment, severe asthmatics reduced, in a significant manner, the number of exacerbations and the eosinophil count, although they did not improve lung function (forced expiratory volume measured during the first second [FEV_1_], and FEV_1_/forced vital capacity [FVC] ratio) neither asthma control test (ACT) values (Table 1). Finally, we observed in the asthmatic population that severe asthmatic patients at baseline had significant higher number of exacerbations, and lower FEV_1_ and ACT values than mild to moderate asthmatics (Table 1).

In view of these data, benralizumab is able to improve asthma clinical parameters in severe eosinophilic patients.

### 3.2. MiRNA Deregulation before and after Benralizumab Administration

**Figure 1:**
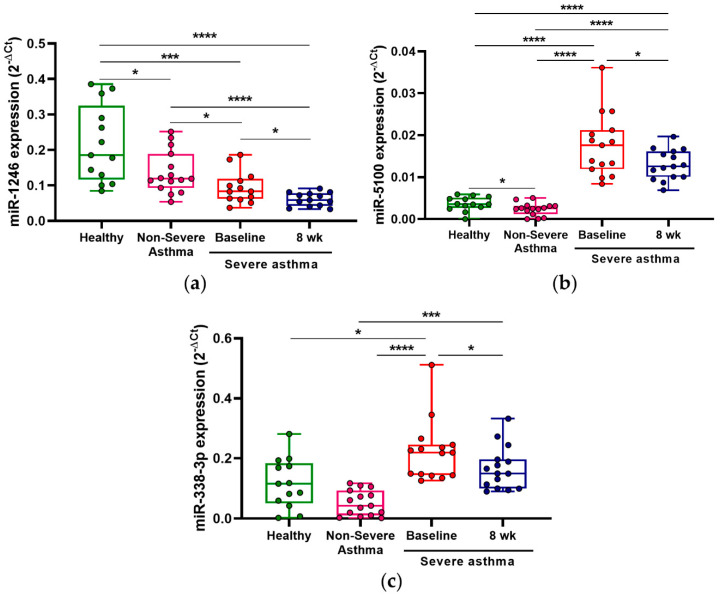
Serum miRNA deregulation in severe patients treated with benralizumab. Severe eosinophilic asthmatic patients showed an altered expression of miR-1246 (**a**); miR-5100 (**b**); and miR-338-3p. (**c**) 8 weeks after benralizumab administration. Also, miR-5100 and miR-338-3p expression levels were higher in severe asthmatics at baseline than in subjects with non-severe asthma, and miR-1246 showed lower levels in this same group of patients. Severe asthmatics at baseline exhibited deregulated levels of these miRNA in comparison to healthy subjects. * *p* < 0.05, *** *p* < 0.001, **** *p* < 0.0001.

In this study, fifteen severe asthmatic patients were recruited and serum miRNAs were evaluated at baseline and eight weeks after the first doses of benralizumab were received. Among the miRNAs checked, we found a significant decrease in the expression levels of three miRNAs after eight weeks of treatment: miR-1246 (0.09 ± 0.04 vs. 0.06 ± 0.02 arbitrary units, *p* < 0.05; Figure 1a), miR-5100 (0.018 ± 0.008 vs. 0.013 ± 0.004 arbitrary units, *p* < 0.05; Figure 1b) and miR-338-3p (0.22 ± 0.10 vs. 0.17 ± 0.07 arbitrary units, *p* < 0.05; Figure 1c). The rest of the miRNAs evaluated did not show any significant differences (Figure S1). and miR-4521 was not detected (data not shown).

Additionally, we evaluated miR-1246, miR-5100 and miR-338-3p expression levels in non-severe asthmatic patients and in healthy non-asthmatic individuals. We observed significantly higher expression levels of miR-5100 and miR-338-3p in severe asthmatic patients with respect to patients with mild-to-moderate asthma (*p* < 0.0001) (Figure 1b,c). On the contrary, the levels of miR-1246 were lower in severe asthmatics at baseline than in non-severe patients, reaching significant differences (*p* < 0.05; Figure 1a). Moreover, the levels of these three miRNAs were significantly deregulated in severe asthmatics at baseline compared to healthy individuals (Figure 1a–c).

These results show that there is an alteration of three miRNAs after benralizumab therapy in severe eosinophilic patients, which could serve as early response markers to treatment.

### 3.3. Deregulated miR-1246 Correlates with Clinical Parameters

**Figure 2:**
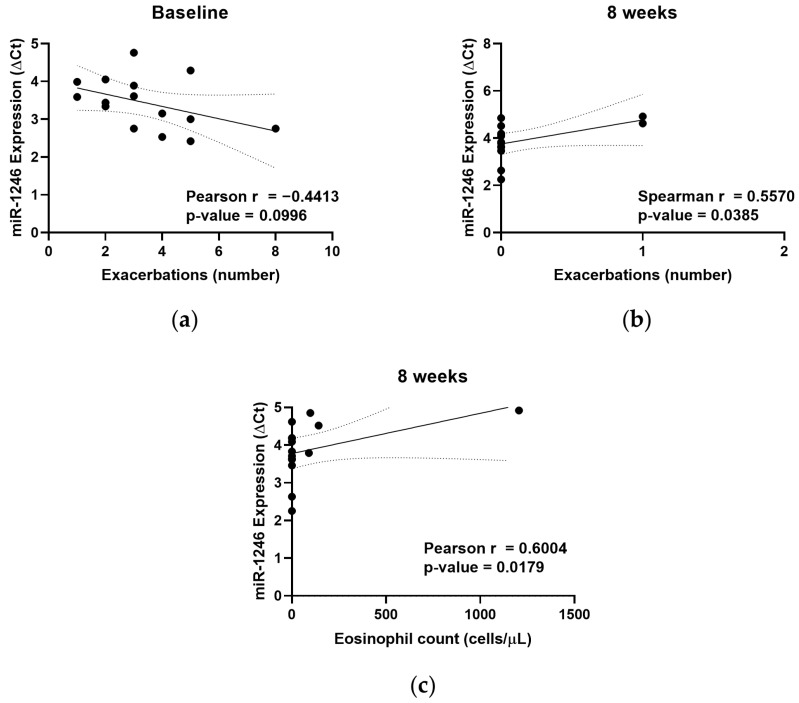
MiR-1246 correlates with the number of exacerbations and eosinophil count. (**a**) Deregulated miR-1246 in severe asthmatic patients at baseline shows a negative weak correlation with exacerbation number. Additionally, positive weak correlations are observed between ΔCt values of this miRNA with the exacerbation number (**b**) and peripheral blood eosinophil count (**c**) in patients with severe asthma 8 weeks after benralizumab administration.

In order to establish some relation between miRNA expression levels and clinical parameters, we performed a correlation analysis. We compared the expression levels of miR-1246, miR-5100 and miR-338-3p with several symptoms, laboratory data and clinical parameters from severe asthmatics before and after benralizumab administration (number of exacerbations, eosinophil count, ACT and FEV_1_). We highlight that ΔCt values of miR-1246 were inversely correlated with the number of exacerbations at baseline (Figure 2a), which means that the number of exacerbations is increased when miR-1246 levels are augmented. Additionally, ΔCt values of this miRNA showed a positive correlation with the number of exacerbations and eosinophil count at eight weeks after treatment (Figure 2b,c), underlying that a higher ΔCt implies a lower expression level. We can highlight that these parameters (eosinophil count and number of exacerbation) were significantly reduced in severe asthmatic individuals after benralizumab therapy (Table 1). Finally, it is worth noting that the main limitation of these analyses is the weakness that the correlations between miR-1246 expression and disease parameters showed (correlation coefficient less than 0.7; Figure 2a–c). In addition, it should be noted that some of the values seem to be outliers, which may obscure the correlation results (Figure 2c). Therefore, the number of measurements should be increased to confirm these results.

On the basis of these findings, we concluded that miR-1246 could be an indicator of patient improvement.

### 3.4. Altered miRNAs are Implicated in MAPK Signaling Pathway

In order to understand the implications on biological processes of miR-1246, miR-5100 and miR-338-3p, an in silico analysis was performed with these three miRNAs. The analysis revealed that the interaction of the three miRNAs, regulated in a significant manner, the mitogen-activated protein kinase (MAPK) signaling pathway (*p* = 0.036). Among the target genes found, there were some relevant genes implicated in asthma pathogenesis, including the nuclear factor of activated T-cells, cytoplasmic 3 (*NFATC3*), mitogen-activated protein kinase kinase kinase 2 (*MAP3K2*), dual-specificity phosphatases family (*DUSP1*, *DUSP2*, *DUSP5* and *DUSP16*) and nuclear factor kappa β subunit 2 (*NFKB2*) (Table 2).

The target genes and signaling pathways that are regulated by these miRNAs could be indicative of a restoration of altered processes in severe asthma after benralizumab treatment.

## 4. Discussion

This is the first report that shows an altered expression of miRNAs (miR-1246, miR-5100 and miR-338-3p) after eight weeks of benralizumab administration, confirming that they could be used as early response markers.

Benralizumab is a monoclonal, afucosylated antibody used as a treatment in severe eosinophilic asthma that binds to IL-5 receptor subunit alpha (IL-5Rα) and depletes, almost completely, peripheral blood eosinophils, being more effective than other anti-IL-5 therapies such as mepolizumab and reslizumab [11,21]. In the studied population, we observed a reduction in the number of exacerbations and eosinophil counts in severe asthmatic patients after benralizumab administration, similar to previous informed clinical trials [8,9].

However, several studies have described the biomarkers for eosinophilic severe asthma, with peripheral eosinophils being considered the best predictor biomarkers for anti-IL-5 and anti-IL-5Rα therapies [22]. However, the more useful biomarkers must be found to predict early response to biological treatment. Thus, we found three miRNAs that could serve this purpose. To date, investigations about this topic are developing, and a current study shows that L-selectin and Krebs von den Lungen (KL-6) are useful novel biomarkers of early response to mepolizumab, but not for benralizumab unfortunately [13]. Thus, more studies in this field should be performed.

According to our results, the altered miRNAs found in patients post-treatment have been linked to or dysregulated in asthma pathogenesis [16,23,24,25]. However, miRNAs have never been described as markers or predictors of early response to biological treatments, as we have done in this report. miR-1246 is probably the most important miRNA in benralizumab response in severe asthmatic patients, because it was significantly correlated with the number of exacerbations per year and with the eosinophil count in these patients, although these correlations were weak (correlation coefficient less than 0.7, Figure 2). Previously, our research group demonstrated altered expression levels of miR-1246 between healthy subjects and asthmatic patients, and it could be used as a diagnosis tool in combination with other miRNAs by a logistic regression model [16]. Additionally, this miRNA has been described as deregulated in other respiratory pathologies such as COPD and asthma-COPD overlap syndrome (ACOS) [26]. Other authors demonstrated that miR-1246 is overexpressed in airway epithelial brushings from asthmatics compared to healthy patients [27], while other studies showed that a module of miRNAs expression in sputum from asthmatics, which included miR-1246 correlated with blood eosinophil count, with the fractional exhaled nitric oxide (FeNO), and with bronchodilator response [28]. As we observed, target genes for this deregulated miRNA belong to MAPK pathways that may regulate cell functions as proliferation or muscle contraction, which are processes that are linked to asthma pathology and its clinical manifestations [29]. In this sense, smooth muscle cells of the lung could be a possible target cell for this miRNA. This fact has been corroborated in other diseases, mainly in cancer, where multiple manuscripts describing the increased expression of miR-1246 in patients with this pathology and its possible role in proliferation and cell migration in colorectal cancer exist [30]. Even miR-1246 could be implicated in atherosclerosis in regards to its relation with vascular smooth muscle cell proliferation [31], with miRNA being a potential therapeutic target in this disease. Less is known about the role of miR-5100 and miR-338-3p in asthma and in other respiratory diseases. While several previous studies have shown these miRNAs to be possible biomarkers in asthma pathology [23,25], no studies on biological treatment response have been developed.

In relation to signaling pathways regulated by these miRNAs, MAPK signaling is essential in the allergic inflammation of airways, and subsequently, in asthma pathogenesis [32]. We found that this pathway was significantly altered by miR-1246, miR-5100 and miR-338-3p, and they regulate some important genes in asthma. DUSP family genes, including *DUSP1*, *DUSP2*, *DUSP5* and *DUSP16*, are the major regulators of the MAPK signaling pathway and they are implicated in the control of anti-inflammatory responses, mainly in References [33,34,35]. A study developed by Kozmus et al. demonstrated that *MAP3K2* was significantly decreased in asthmatic patients after ICS treatment [36]. This fact could be indicate that the alteration in gene expression could occur via miRNA regulation, although more studies must be developed to elucidate it. Finally, *NFATC3* and *NFKB2* play a central role in cytokine regulation in several types of immune cells [37,38]. One miRNA could simultaneously affect and multiply genes. In this sense, although some genes of the MAPK signaling pathway could be affected by the deregulated miRNAs observed in our study, not all of them could be implicated in pro-inflammatory mechanisms, and these miRNAs could exert different regulatory effects on different genes. We can speculate that compensatory mechanisms could exist; thus, these miRNAs could trigger inflammatory processes by other pathways. Perhaps the miRNA deregulation before and after treatment in severe asthmatic patients has the objective of recovering the normal function of this pathway and the correct expression of these target genes in order to reestablish the control of asthma mechanisms. However, the specific role of these miRNAs in benralizumab response should be studied more extensively.

## 5. Conclusions

Here, we report, for the first time, three miRNAs that could be used as biomarkers of early benralizumab response in severe eosinophilic asthmatic patients. The downregulation of miR-1246, miR-5100 and miR-338-3p eight weeks after the first benralizumab dosage could indicate a recovery of control mechanisms of asthma in these patients, and subsequently, an improvement of their health.

## Figures and Tables

**Table 1:** Clinical and demographic characteristics of the studied patients.

	Asthma (Mild-Moderate) (*n* = 15)	Asthma (Severe)		*p*-Value
		Baseline (*n* = 15)	8 Weeks (*n* = 15)	
Age (years) ^a^	41.20 ± 8.75	46.86 ± 11.48		***
Male (%)	3 (20)	6 (40)		N.S.
Eosinophils (cell/μL)	300 (200–500)	217 (91–625)	0 (0–90)	N.S./****/^‡^
FEV_1_ (%) ^a^	97.99 ± 11.70	71.00 ± 16.56	74.08 ± 17.59	***/****/N.S.
FEV_1_/FVC (%) ^a^	N.A.	63.56 ± 10.16	63.11 ± 9.89	N.S.
Exacerbation per year ^b^	1 (0–1.25)	3 (2–5)	0 (0–0)	***/*/^†^
ACT	23 (21–25)	11 (9.5–14)	13 (10.5–24.5)	***/N.S./N.S

^a^ Results are expressed as mean ± SD. ^b^ Results are expressed as median (IQR). Comparisons were performed in the next order: Asthma vs. Baseline/Asthma vs. 8 weeks/Baseline vs. 8 weeks. Comparisons between Asthma and Severe Asthma are represented with asterisk: * *p* < 0.05, *** *p* < 0.001, **** *p* < 0.0001. Comparisons between Baseline and 8 weeks are represented with symbols: ^‡^ *p* < 0.001, ^†^ *p* < 0.001. ACT, asthma control test; FEV_1_, forced expiratory volume measured during the first second; FVC, forced vital capacity; N.A., not available; N.S., non-significant.

**Table 2:** Target genes of miR-1246, miR-5100 and miR-338-3p implicated in the MAPK signaling pathway.

miRNA	Pathway	Target Genes
miR-1246	MAPK signaling pathway	*TAOK1*, *TP53*, *PPP3CA*, ***MAP3K2***, *RASGRP3*, ***NFATC3***
miR-5100		*TAOK1*, *PLA2G4A*, ***DUSP16***
miR-338-3p		*FOS*, *CACNG8*, ***DUSP2***, ***DUSP5***, *ELK4*, *CDC25B*, *TAOK2*, *MAP2K3*, *MAP4K3*, *RASA1*, *RAPGEF2*, ***NFKB2***, *MAPKAPK3*, *ZAK*, *HSPA8*, *CACNA1H*, ***MAP3K2***, *RPS6KA4*, ***NFATC3***, ***DUSP1***

## Data Availability

The data presented in this study are available on request from the corresponding author. The data are not publicly available due to it includes personal data of patients.

## Supplementary Materials

The following are available online at https://www.mdpi.com/2075-4426/11/2/76/s1, Figure S1: Serum miRNAs levels evaluated in severe eosinophilic asthmatic patients before and after benralizumab injection.

## Abbreviations

ACOS	Asthma-COPD overlap syndrome
ACT	Asthma control test
ADCC	Antibody-dependent cell-mediated cytotoxicity
ATS	American Thoracic Society
COPD	Chronic obstructive pulmonary disease
Ct	Cycle threshold
ERS	European Respiratory Society
FeNO	Fractional exhaled nitric oxide
FEV_1_	Forced expiratory volume measured during the first second
FVC	Forced vital capacity
GINA	Global Initiative for Asthma
ICS	Inhaled corticosteroids
IL-5Rα	IL-5 receptor subunit alpha
IQR	Interquartile range
LABAs	Long-acting β2-agonists
MAPK	Mitogen-activated protein kinase
miRNAs	MicroRNAs
SD	Standard deviation
