# Supplementary material for: Serum microRNAs as Tool to Predict Early Response to Benralizumab in Severe Eosinophilic Asthma
Source: J Pers Med. 2021 Jan 28;11(2):76. doi: 10.3390/jpm11020076 (PMC7912443; doi:10.3390/jpm11020076)
Supplement: Supplementary file 1 [file jpm-11-00076-s001.pdf]

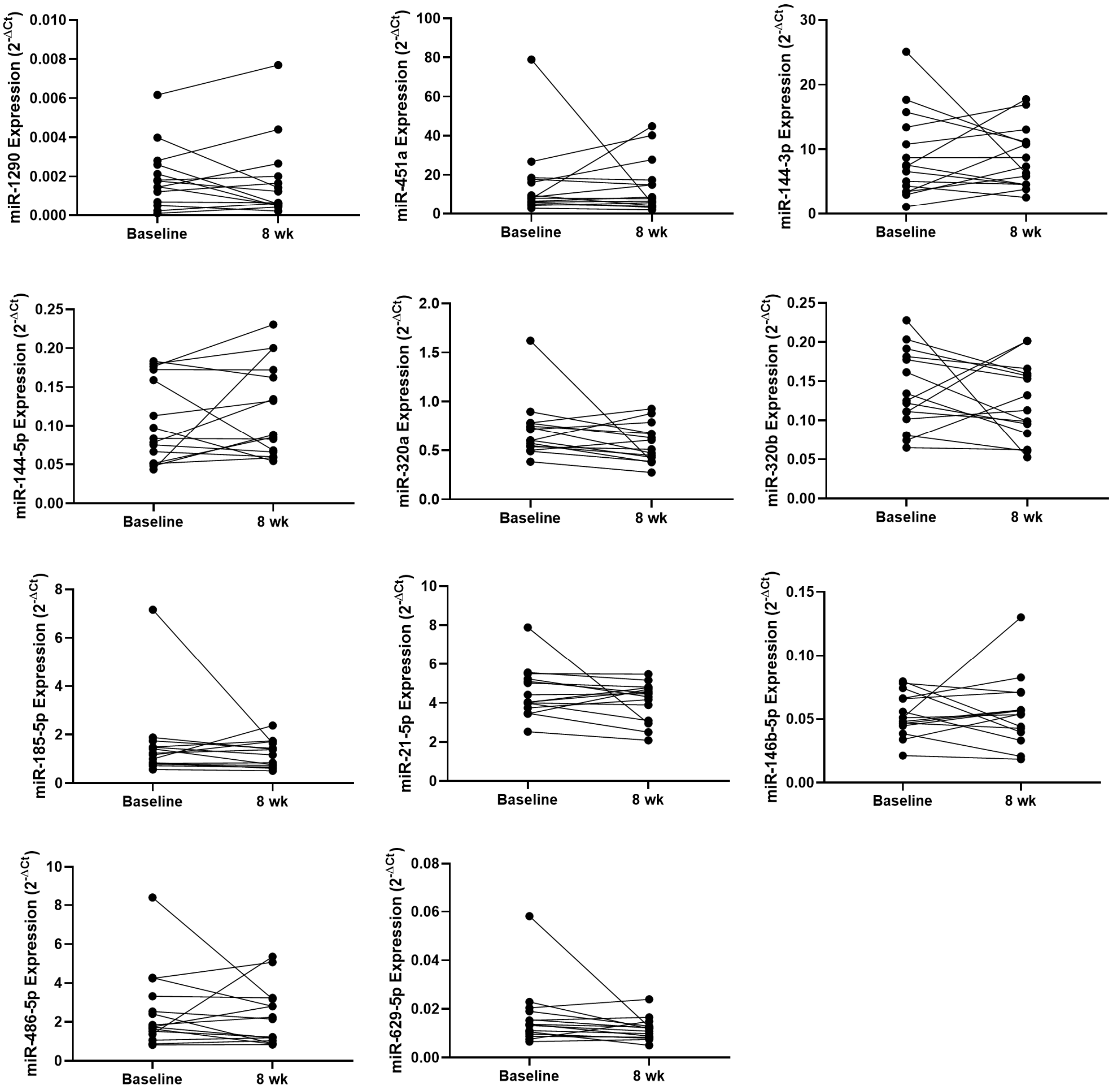

Figure S1: Serum miRNAs levels evaluated in severe eosinophilic asthmatic patients before and after benralizumab injection.
